# Electrochemical-Based Sensing Platforms for Detection of Glucose and H_2_O_2_ by Porous Metal–Organic Frameworks: A Review of Status and Prospects

**DOI:** 10.3390/bios13030347

**Published:** 2023-03-04

**Authors:** Hessamaddin Sohrabi, Fatemeh Maleki, Pegah Khaaki, Mohammed Kadhom, Nurbolat Kudaibergenov, Alireza Khataee

**Affiliations:** 1 Department of Analytical Chemistry, Faculty of Chemistry, University of Tabriz, Tabriz 51666-16471, Iran; 2 Department of Biology, Tabriz Branch, Islamic Azad University, Tabriz 51666-16471, Iran; 3 Department of Environmental Science, College of Energy and Environmental Science, Alkarkh University of Science, Baghdad 10081, Iraq; 4 Department of Chemistry and Chemical Technology, Al-Farabi Kazakh National University, Al-Farabi 71, Almaty 050038, Kazakhstan; 5 Research Laboratory of Advanced Water and Wastewater Treatment Processes, Department of Applied Chemistry, Faculty of Chemistry, University of Tabriz, Tabriz 51666-16471, Iran; 6 Department of Environmental Engineering, Faculty of Engineering, Gebze Technical University, 41400 Gebze, Turkey

**Keywords:** MOF-based nanocomposites, porous materials, sensing assays, electrochemical techniques, H_2_O_2_ determination

## Abstract

Establishing enzyme-free sensing assays with great selectivity and sensitivity for glucose and H_2_O_2_ detection has been highly required in biological science. In particular, the exploitation of nanomaterials by using noble metals of high conductivity and surface area has been widely investigated to act as selective catalytic agents for molecular recognition in sensing platforms. Several approaches for a straightforward, speedy, selective, and sensitive recognition of glucose and H_2_O_2_ were requested. This paper reviews the current progress in electrochemical detection using metal–organic frameworks (MOFs) for H_2_O_2_ and glucose recognition. We have reviewed the latest electrochemical sensing assays for in-place detection with priorities including straightforward procedure and manipulation, high sensitivity, varied linear range, and economic prospects. The mentioned sensing assays apply electrochemical systems through a rapid detection time that enables real-time recognition. In profitable fields, the obstacles that have been associated with sample preparation and tool expense can be solved by applying these sensing means. Some parameters, including the impedance, intensity, and potential difference measurement methods have permitted low limit of detections (LODs) and noticeable durations in agricultural, water, and foodstuff samples with high levels of glucose and H_2_O_2_.

## 1. Introduction

Glucose is the main energy supply source in organisms, and changes in the standard levels of this substance, low or high, may result in diseases [1]. By considering glucose ranges in the blood, diabetes signals in humans can be managed and controlled [2]. Today, diabetes has become a common, chronic, and global health burden disease with fatal consequences [3]. When contrasted with other diseases, it may result in complications such as renal failure, blindness, stroke, and cardiovascular disease. Since diabetes is a chronic disease, monitoring the concentration of blood glucose repeatedly could facilitate the diabetic lifestyle and improve treatment efficiency [4]. A typical reactive oxygen species (ROS), namely, hydrogen peroxide (H_2_O_2_), has a major role in the foodstuff industry and several normal biological functions [5]. H_2_O_2_ is mostly derived from protein folding, cell respiration, metabolic metabolisms in organisms, and regulated biochemical reactions in animals and plants. The normal H_2_O_2_ level in organisms guarantees the signal transduction and regular physiological function of cells [6]. H_2_O_2_, a valued oxidant, has widely been employed in different fields, such as the chemical and foodstuff industries, papermaking, textiles, wastewater treatment, and medical equipment [7]. An unbalanced level of H_2_O_2_ may result in human diseases including cardiovascular diseases [8], atherogenesis [9], aging [10], and neurodegenerative Alzheimer’s [11]. The importance of glucose and H_2_O_2_ in several biological fields presents the requirement for a rapid and sensitive detection method [12]. The efficient recognition of glucose and H_2_O_2_ has received increased attention because of their significant roles in the foodstuff industry and clinical diagnostics. Glucose and H_2_O_2_ concentrations in biological bodies are highly associated with physiological health. The immediate recognition of glucose and H_2_O_2_ in biological bodies is advantageous to find out the relationship of inherent small molecule-disease; in fact, on-time and accurate diagnosing of diseases is important in monitoring the progression of the disease [13].

With the aim of speedy diagnosis of diabetes and immediate monitoring of sugar levels in the blood, many sensors have been explored and established [14]. Hence, acoustic, electrochemical, transdermal, optical, and fluorescent techniques were effectively used to monitor glucose levels. On the other hand, the disadvantages of these techniques, such as the long detection period and costly tools, should not be ignored [15]. In the past few decades, numerous techniques, including electrochemical sensors, fluorescence, calorimetry, and gas chromatography, have been applied to recognize glucose concentration in foodstuff products and human serum. Among the mentioned techniques, gas chromatography and calorimetry are time-consuming ones that necessitate intricate preprocessing along with a professional information background, whereas fluorescence can effortlessly be disturbed through interfering substances [16]. More recently, several methods have been developed to assess the H_2_O_2_ content, but many of them have disadvantages, including high cost, low selectivity, and being time-consuming [17]. Among the currently developed analytical techniques, the electrochemical ones display the advantages of rapid response, easy process, sensitivity, and low cost; therefore, they are preferably considered [18]. To avoid the disadvantages of clinical approaches, we inevitably moved on to new methods of sensing platforms.

**Scheme 1:**
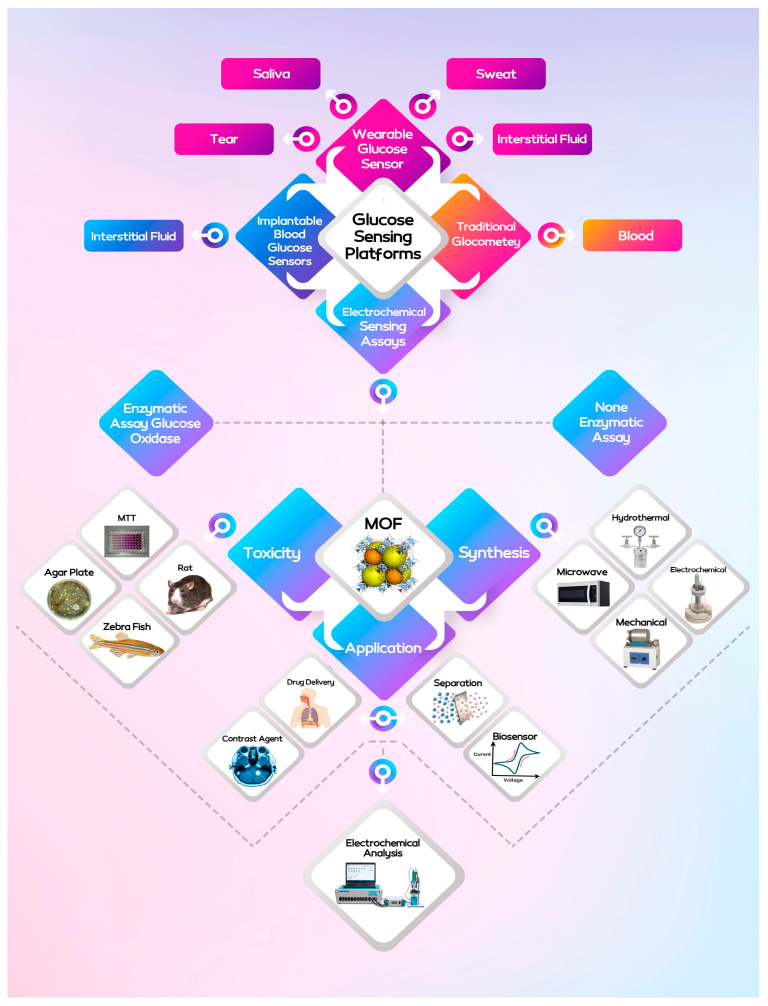
Graphical representation of the MOF-based sensing platforms for determination of glucose.

Electrochemical sensors and biosensors have been extensively applied in analytical chemistry [19], clinical diagnosis [20], medical analysis [21], and food detection [22] owing to their hypersensitivity, immediate monitoring, low cost, straightforward preparation process, and selectivity. Electrochemical sensors are divided into two groups: (i) enzyme biosensors and (ii) non-enzyme biosensors. Formerly, enzyme-based biosensors were more common thanks to their high sensitivity and selectivity. At present, non-enzymatic electrochemical sensors are demanding significant attention for applied usage compared with enzymatic biosensors because denaturation can simply degrade the biological efficiency of enzymatic biosensors [17]. The progress of novel electrochemical sensors has been an unceasingly increasing field in electrochemistry and nanomaterials. However, the growing demands for precise sensing have considerably developed the design and construction of functional materials. Many inorganic materials, such as silica nanoparticles (NPs), metallic NPs, MOFs, graphene oxide (GO), and quantum dots, as well as organic materials are being applied as platforms in sensing assay design. Among the mentioned ones, MOFs with particular functional sites are demanding much attention in developing sensing and biosensing platforms as a result of their dissimilar structures and multi-functionalities, enabling specific molecular detection [23]. Hence, the most recent development of MOFs made exceptional accomplishments in the electrochemical recognition of glucose, H_2_O_2_, and heavy metal ion sensing [24]. In this review, MOFs’ general description, assembly, and high-potential employment are demonstrated. In the following, the importance of glucose and H_2_O_2_ detection will be emphasized (Scheme 1). Finally, MOF-based electrochemical sensing platforms that have been developed for a considerable period, along with prior research works associated with glucose and H_2_O_2_ detection, will be presented to discuss their obstacles and upcoming perspectives.

## 2. MOFs: General Definition and Structure

MOFs are a new kind of microporous compound fabricated from metallic modes that have been connected through organic ligands [25]. The metallic content of inorganic nodes in MOFs represents active sites that play a significant role in assessing their catalytic function [26]. MOFs have received attention because of their high surface areas, highly variegated assembly, customizable chemistry, tunable pore structural design, various structures, ultra-low densities, thermal stability, and simple synthesis routes suitable for chemical and physical applications [27]. They have been extensively employed in the areas of drug delivery, catalysis, gas separation/adsorption, energy storage, and sensors as a result of the aforementioned benefits [28]. MOFs, also known as porous coordination polymers, are 3D-ordered porous materials which, in certain cases, exhibit record-setting internal surface areas [29]. Based on these exclusive properties, the redox-active MOFs demonstrate high potential in electrochemical applications and energy storage. Furthermore, the redox-active activity of MOFs can possibly be applied as an electrochemical sensing stand [30].

Topologically, all MOFs are formed from secondary building units (SBUs) constructed from oxygen atoms and metal ions. Furthermore, organic linkers play a major role in connecting the SBUs within MOFs’ framework. The progress of MOFs brought significance to the structural scope since they were assembled via different shapes (squares, triangles, tetrahedrons, and octahedrons) SBUs. Every year, several types of SBUs are attached to organic linkers to synthesize thousands of new MOFs [31]. To modulate chemical functionalities and the crystalline structure of MOFs, inorganic and organic components must be selected wisely [32]. The metallic components of MOFs, metal clusters, and/or isolated metal centers, participate in its catalytic activity. Incorporating MOFs with one or more additional metal centers can increase their specified function [33]. Considering their ultimate properties and structures, MOFs are possibly manufactured via a diverse range of synthetic techniques, such as mechanochemical [34], hydrothermal (solvothermal) [35], slow diffusion [36], electrochemical [37], microwave-assisted heating, and ultrasound [38]. MOFs are prepared in different forms, including nanoparticles, nanospheres, nanorods, and nanosheets.

## 3. Electrochemical-Based Sensing Assay for Detection of Glucose

In recent years, electrochemical approaches provided an enlarged potential for the on-site detection of materials through improving speedy analysis and proper miniaturization. Currently, electrochemical methods are increasingly prevalent in sensing studies in comparison with other detective tools, such as spectro-photometric colorimetric and surface plasmon resonance methods. Recently, electrochemical platforms were rapidly established and are used due to their speedy sensing time, high sensitivity, straightforward process, and low cost [39]. Different electrochemical approaches of voltammetric, electrochemiluminescence, amperometric, and impedimetric were employed for this purpose [39]. Nanomaterial-based compounds in electrochemical sensing tools have a promising prospect as a result of their biocompatibility and preserving biological capability during absorption [40]. The overall electrochemical sensing mechanism of glucose is based on glucose oxidation to gluconolactone by applying the reductive form of the electroactive synthesized MOF-based compounds. For instance, Co (III), Cu (III), and Ni (II) are formed on the surface of the nanoparticles. Meanwhile, the oxidation peak current increases, whereas the reduction peak current decreases, owing to the feeding of Co (III), Cu (III), and Ni (II) agents and the formation of Co (II), Cu (II), and Ni agents. The electroactive MOF-based compounds and glucose molecules undergo an electrochemical reaction, releasing electrons and producing electrical signals proportional to the glucose concentration. Table 1 demonstrates various electrochemical-based detecting platforms for the highly sensitive determination of glucose.

Wei et al. [41] used NiMn layered double hydroxide (LDH)-based MOF (NiMn-LDH-MOF) material as electrochemical electrode in a sensitive assay of glucose. The NiMn-LDH-MOF was synthesized by one-pot method via directly blending MOF-74, metal nodes, and ammonia. The obtained NiMn-LDH-MOF has excellent electrocatalytic activity for electrochemical oxidation of glucose and the NiMn-LDH-MOF/GCE sensor exhibits a wide linear detection range from 4.9 μM to 2.2 mM, a low detection limit (0.87 μM, S/N = 3), and high sensitivity (0.849 mA mM^–1^ cm^–2^). Meanwhile, the sensor has good selectivity and reliability for glucose detection in real serum samples, suggesting the potential applicability of the sensor for glucose detection. The more exposed active sites, the introduction of LDHs subunit, and the synergistic effect between Ni and Mn in the NiMn-LDH-MOF are the major factors enhancing the assay performance.

Zeraati et al. [42] established a Ni-MOF electrochemical sensor for glucose recognition. The MOF was manufactured with a mean particle size of 80 nm, significant porosity of 1.14–9.6 nm, and a surface area of 1381 m^2^/g. The amperometric response of the electrode toward glucose was achieved at a steady state in 3 s. The LOD, linear range, and sensitivity for glucose were 0.76 µM (S/N = 3), 1–1600 µM, and 2859.95 µA mM^−1^ cm^−2^ (R^2^ = 0.9966), respectively. In comparison with non-enzymatic ones, the provided electrode has promising standards to develop a sensor of higher sensitivity and lower LOD. In another work performed by Liu et al. [1], Cu has been doped into a Co-based MOF of a 2D structure to detect glucose and oxygen evolution reaction (OER). Two-dimensional book-like CuCo-MOF was grown in situ on carbon fiber paper (CFP) by a one-step solvothermal reaction, which can be directly used as a general anodic electrocatalytic material for glucose detection and water oxidation. The electrocatalytic glucose recognition had a linear range, high sensitivity, and a low detection limit of 10–1200 µM, 6.861 mA mM^−1^ cm^−2^ and 0.12 µM, respectively. For the OER, once the density reached 10 mA cm^−2^, the overpotential (Z) was 340 mV, and well stability was preserved for approximately 10 h without current weakening. The 2D MOF structure had an increased potential in the electrocatalytic OER.

**Figure 1:**
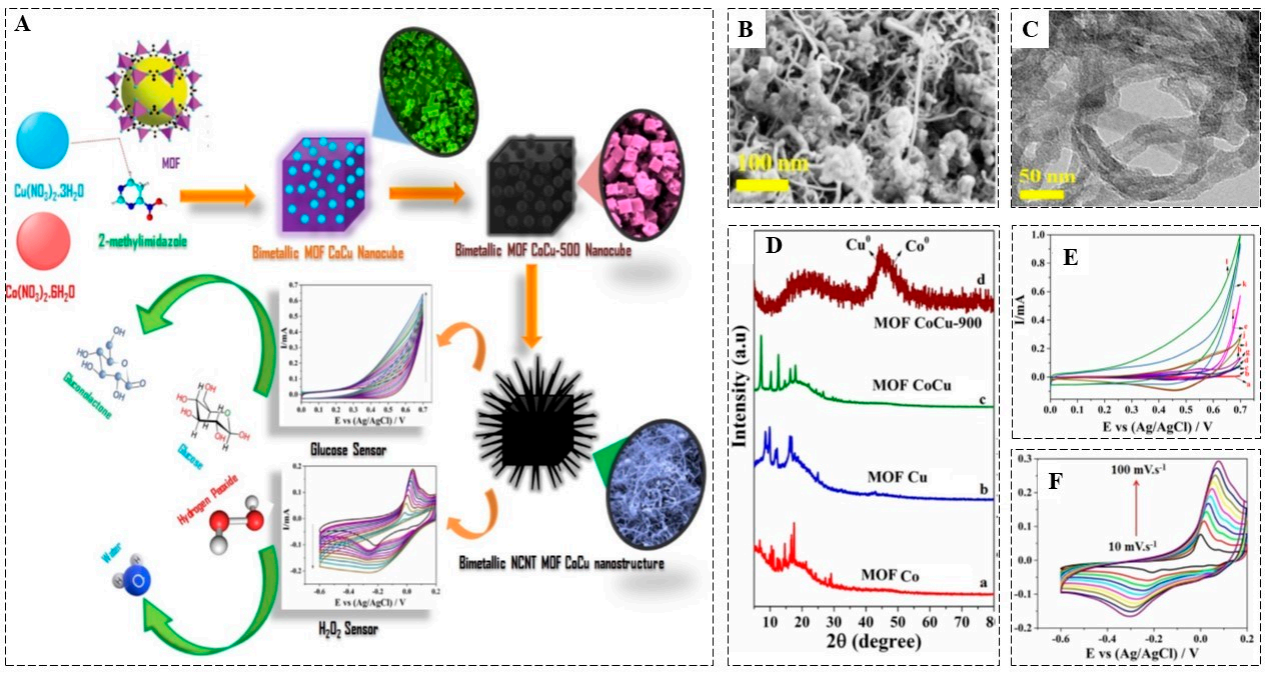
(**A**) Graphic diagram of the bimetallic nanostructure of the synthesized MOF for electrochemical H_2_O_2_ and glucose sensing, (**B**) FE-SEM image of bimetallic nanostructure of NCNT MOF CoCu, (**C**) TEM image, (**D**) XRD schemas of (a) Co MOF, (b) Cu MOF, (c) CoCu MOF nanocubes, and (d) the prepared bimetallic nanostructure of MOF, (**E**) Cyclic voltammograms of (a, b) unmodified GCE, (c, d) Cu MOF, (e, f) Co MOF, (g, h) nanocube structures of CoCu MOF, (i, j) CoCu-500 MOF, and (k, l) the synthesized nanostructure of bimetallic CoCu MOF, and (**F**) Cyclic voltammograms of the bimetallic CoCu MOF. Adapted by permission from Ref. [43].

A hierarchical 3D nitrogen-doped carbon nanotube anchored bimetallic cobalt copper organic framework (NCNT MOF CoCu) has efficaciously been fabricated through the direct growth method by applying high-temperature carbonization of bimetallic cobalt copper organic structure (MOF CoCu-500) by applying 2-methylimidazole as an effective ligand (Figure 1A). High-level function for H_2_O_2_ and glucose molecules sensing is possessed by the NCNT MOF CoCu nanostructure. The presence of nitrogen-doped carbon in nanotube array form immobilized on the surface of MOF CoCu nanostructure is effectively confirmed by applying the TEM and FE-SEM as depicted in Figure 1B,C. On the other hand, Figure 1D demonstrates the XRD schemas of (a) Co MOF, (b) Cu MOF, and (c) nanocube structures of CoCu MOF. Moreover, to explain the successful fabrication of Cu and Co nanoparticles, the XRD pattern of NCNT MOF CoCu nanostructure is demonstrated in Figure 1D(d). The typical peaks associated with XRD patterns placed at 2θ = 43.7° and 45.6° are apportioned to diffraction planes of cubic copper and cobalt, respectively. For electrochemical behavior investigation, Figure 1E explains the cyclic voltammetry (CVs) of (a, b) unmodified GCE (glassy carbon electrode), (c, d) Cu MOF, (e, f) Co MOF, (g, h) nanocube structures of CoCu MOF, (i, j) CoCu-500MOF, and (k, l) the prepared bimetallic NCNT CoCu MOF. Figure 1F shows cyclic voltammograms of the nanostructure related to the bimetallic NCNT CoCu MOF. This research presented a first-rate electrocatalytic presentation for glucose oxidation with a linear range of 0.05 to 2.5 mM, a sensitivity of 1027 μA mM^−1^ cm^−2^, and an LOD of 0.15 μM. In the same way, the NCNT MOF CoCu nanostructure had considerably higher H_2_O_2_ function with a linear range, sensitivity, and detection limit of 0.05 to 3.5 mM, 639.5 μA mM^−1^ cm^−2^, and 0.206 μM, respectively. The bimetallic NCNT MOF CoCu nanostructure modified GCE current response retains at least 96% of the original value after 60 days. These results are owed to the synergistic effect between NCNT and bimetallic CoCu in the organic structure. This structure gives highly graphitized carbon, homogeneous nitrogen-doped carbon nanotubes, and a unique hierarchical nanoarchitecture. In addition, as an appropriate probe for H_2_O_2_ and glucose detection, the fabricated sensor has been applied in real models [43].

**Figure 2:**
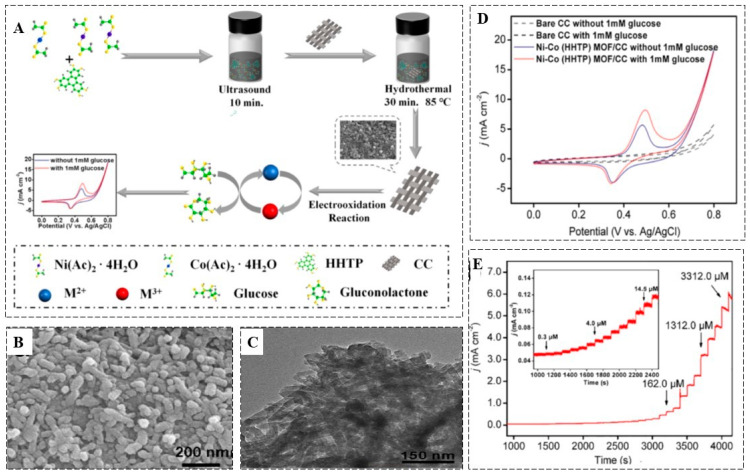
(**A**) Synthetic progression of the MOF and its usage in the determination of glucose, (**B**) The SEM image of Ni/Co(HHTP)MOF/CC, (**C**) The TEM image of the synthesized MOF, (**D**) CVs of the unmodified carbon cloth and the fabricated Ni/Co(HHTP)MOF, and (**E**) Amperometric responses. Adapted by permission from Ref. [16].

Xu et al. [16] set up an enzyme-free electrochemical-based glucose sensor by applying direct growth of a conductive bimetal by applying HHTP (HHTP = 2,3,6,7,10,11-hexahydroxytriphenylene) as an organic linker (Ni/Co(HHTP)) MOF on carbon cloth [Ni/Co(HHTP)MOF/CC] using the hydrothermal technique (Figure 2A). The mentioned figure schematically demonstrates that the direct sensing mechanism is based on the glucose and gluconolactone electrochemical reaction by considering the reductive form of the electroactive synthesized MOF. The surface area and the pore volumes of the bimetallic MOF were 180.3159 m^2^/g and 0.39 cm^3^/g. This improvement was attributed to the first-rate conductivity between Ni/Co(HHTP)MOF and CC, and the synergic catalytic effect of Ni and Co elements. Additionally, Figure 2B displays that it is shaped by firmly linked units like thick rods with a size of one hundred nanometers. This construction owns a greater surface area and more adsorption sites for highly sensitive determination of glucose sensing than that of the smooth carbon cloth. Many unsettled nanorods could be recognized from the TEM image (Figure 2C). On the other hand, Figure 2D displays the CVs of the bare CC and the Ni/Co(HHTP)MOF/CC (the ratio of nickel to cobalt is 2:1) with or without 1.0 mM glucose. Electrochemical responses have indicated that the unmodified CC displays that no redox peak seemed apparent in the absence of glucose, and the CV of the bare CC was hardly altered after the addition of glucose, which confirms that the unmodified CC is comparatively inactive in the glucose redox reaction. Furthermore, Figure 2E confirms the amperometric signals of the Ni/Co(HHTP)MOF. In optimum conditions, the Ni/Co(HHTP)MOF/CC has demonstrated high activity with a linear range, low LOD, sensitivity, and fast response of 0.3 μM-2.312 mM, 100 nM (S/N = 3), 3250 μA mM^−1^ cm^−2^, and 2s, respectively.

Recently, Wang et al. [44] successfully provided an efficient modifier agent based on carbon cloth, where a 3D self-supporting NiO-MOF, which used 2,5-dihydroxyterephthalic acid as an organic linker (MOF-74) and NiCo LDH (layered double hydroxide) composite, was prepared. Straightforward electrochemical deposition and hydrothermal processes were used in the synthesis. By applying the unique flower-like 3D framework, the modified electrode showed appropriate electrochemical behavior, where the capacitance and high rate capability were 9.73 F cm^−2^ of 77.65%, respectively. A high density of 50 mA cm^−2^ and a capacitance retention rate of 84.09% were achieved after 5000 cycles. In addition, the synthesized tool demonstrated superb power and energy density of 1750 W kg^−1^ and 22.85 Wh kg^−1^, respectively. Furthermore, the fabricated nanocomposite presented a first-rate electrocatalytic performance in detecting glucose, with a linear range of 10 μM–1.1 mM, a low detection limit of 278 nM (S/N = 3), and a high sensitivity of 1699 μA mM^−1^ cm^−2^. However, the modified electrode presented acceptable long-term stability after 35 days, which could be assigned to the high surface area and enhanced conductivity. Here, additional active sites for electrochemical reactions were offered and the ion transport direction was reduced by the synergistic effect of the bimetal Ni-Co. Therefore, the constructed CC@MOF-74(NiO)@NiCo LDH may have a potential application point of view in the area of non-enzymatic glucose sensors and supercapacitors.

**Figure 3:**
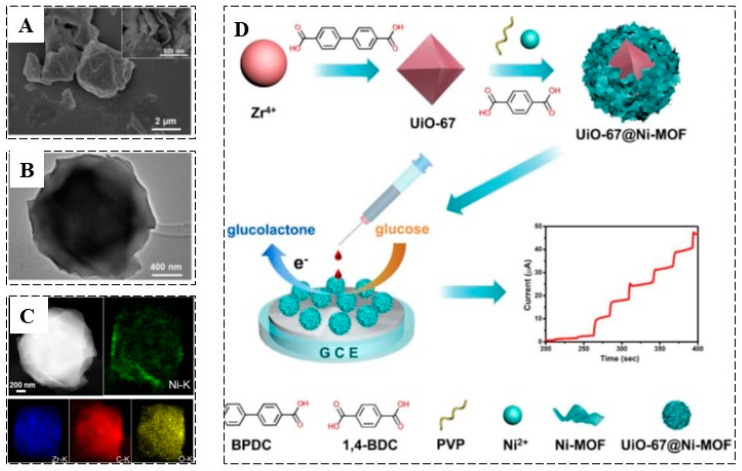
(**A**) SEM, (**B**) TEM images, (**C**) EDS of UiO-67@Ni-MOF, and (**D**) Graphic representation of the fabricated UiO-67@Ni-MOF composite. Adapted by permission from Ref. [46].

Qiaoand et al. [45] presented the application of a conductive Ni-MOF, which is an on-noble-metal catalyst for proficient glucose electrooxidation in alkaline electrolytes. For glucose detection, this Ni-MOF demonstrated the LOD, stability, and sensitivity of 0.66 µM (S/N = 3), 30 days, and 21,744 µA mM^−1^ cm^−2^, respectively, with a rapid response period of lower than 3 s. In addition, the conductive Ni-MOF exhibits a detection range from 0.001 to 8 mM. On the other hand, Lu and coworkers [46] defined core-shell UiO-67@Ni-MOF composites based on an electrochemical sensing platform to detect glucose through a non-enzymatic process. The fabricated core-shell materials based on Ni-MOF (nickel metal structure with 2,5-dihydroxyterephthalic acid as the ligand) and UiO-67 (Zr(IV) biphenyl dicarboxylate metal-organic framework) were fabricated by increasing the internal growth of Ni-MOF shell on a core of UiO-67 through polyvinylpyrrolidone (PVP) direction. By applying this sensing assay, UiO-67 with acceptable conductivity and a huge specific surface area was applied to accelerate the electron transport rate of the prepared nanocomposite. The synergistic effects of the electrocatalytic function of Ni-MOF and the conductivity of UiO-67 enabled glucose detection of the fabricated composite. Specification of electrochemical performance through the CV process revealed that the fabricated electrochemical sensor presented a good electrocatalytic function for glucose oxidation in alkaline environments. The surface morphology of UiO-67@Ni-MOF has been investigated by SEM. Figure 3A illustrates that the composite has a 3D structure with good dispersity. The TEM image of UiO-67@NiMOF reveals that the composite contains Ni-MOF nanosheets as the shell (light areas), and also an octahedral UiO-67 core (dark areas) on the UiO-67 surface (Figure 3B). Moreover, energy-dispersive X-ray spectrometry (EDS) elemental mapping (Figure 3C) reveals that elements including Ni, Zr, C, and O are present and distributed in homogeneous form. On the other hand, Figure 3D illustrates a graphical presentation of the related core-shell composite synthesis. The outcomes at optimum conditions indicate that the sensor contains a fast response (<5 s), LOD (0.98 µM), and an extensive linear range (5 µM to 3.9 mM). Additionally, this sensor has long-range stability, good repeatability (RSD = 1.9%), and reproducibility (RSD = 1.1%). Once the glucose LODs were applied in human serum models, this sensor was efficient in glucose recognition.

**Figure 4:**
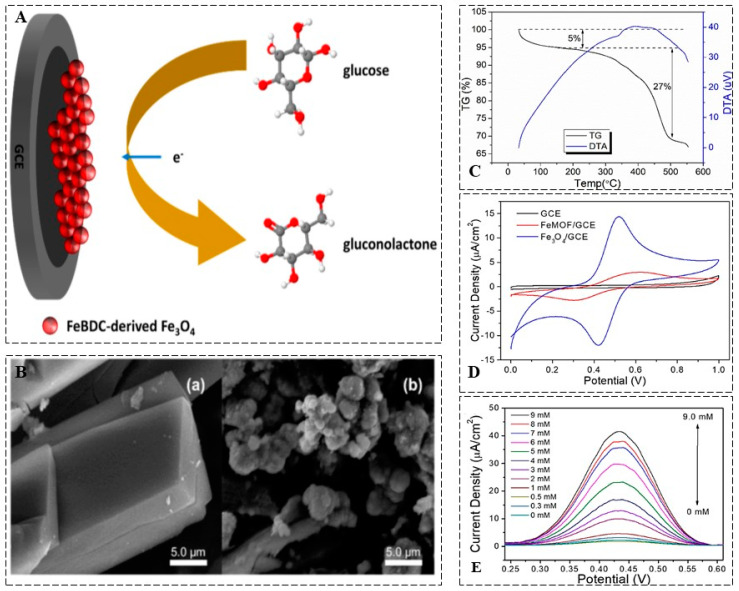
(**A**) Glucose oxidation on the Fe_3_O_4_/GCE, (**B**) SEM images of (a) FeBDC (b) FeBDC-derived Fe_3_O_4_, (**C**) TG/DTA of MOF FeBDC, (**D**) Cyclic voltammogram spectra of bare GCE, FeBDC/GCE. Fe_3_O_4_/GCE, and (**E**) Differential pulse voltammetry measurement of glucose. Adapted by permission from Ref. [48].

Furthermore, Wei et al. [47] have synthesized Co_3_O_4_, CuO, and Cu_2_O nanorod arrays on Cu foam of a seaweed-like structure through stages of in situ growth including alkaline oxidation of CF, solvothermal reaction of Cu(OH)_2_ precursor, and annealing CoCu-MOF. Structure measurements displayed that the Cu(OH)_2_ nanorod arrays offered a stable template for the growth of CoCu-MOF nanosheets, inhibiting the aggregation of MOFs. After annealing, CoCu oxides kept the morphology of nanorod arrays without any collapse and a porous structure was obtained. The prepared CoCu oxide/CF was directly used as a non-enzymatic glucose-sensing electrode without an additional fixed procedure. Through applying the fabricated electrode (CoCu-MOF/Cu_2_O/CFE), high sensitivity of 11.916 μA mM^−1^ cm^−2^, a varied linear range of 0.001–1.07 mM, and detection limit of 0.72 µM have been obtained. The detection performance is related to the synergistic effect of metal oxides’ high electrocatalytic function on binder-free electrodes and the appropriate catalytic sites of porous nanorod array morphology. In addition, acceptable feasibility and selectivity in human serum, first-rate stability, and reproducibility have also been achieved by applying CoCu oxides/CF electrodes, signifying that this may be promising for practical applications. Additionally, Abrori et al. [48] have provided FeBDC-derived Fe_3_O_4_ MOF by the solvothermal process that was used for non-enzymatic electrochemical glucose recognition (Figure 4A). Benzene-1,4-dicarboxylic acid (H_2_BDC) and FeCl_2_.4H_2_O have been applied in place of precursors to form FeBDC. The final MOF has micro-rod-like morphology and crystallinity. Figure 4B shows SEM images of (a) FeBDC and (b) FeBDC-derived Fe_3_O_4_, which shows an amorphous nature of Fe_3_O_4_ with a strongly defective surface. In addition, the thermogravimetry/differential thermal analysis (TG/DTA) curve of MOF FeBDC was presented in Figure 4C. CV and differential pulse voltammetry (DPV) of bare GCE, FeBDC/GCE, and Fe_3_O_4_/GCE were used in glucose determination as shown in Figure 4D,E. Electrochemical traits have been analyzed by treating DPV and CV with a 0.1 M PBS of a pH 7.4 as a supporting electrolyte. The oxidation and reduction peaks in the CV curve of FeBDC and Fe_3_O_4_ were demonstrated by monitoring the outcomes. The reduction and oxidation current peaks increased by pyrolyzing FeBDC to Fe_3_O_4_. The Fe_3_O_4_ sample had a sensitivity of 4.67 µA mM^−1^ cm^−2^, a linear range between 0.0 to 9.0 mM, and a glucose LOD of 15.70 µM. In addition, the current peaks obtained in the five repeated measurements of four independent electrodes showed relative standard deviance of 1.60%, confirming that the results are reproducible.

**Figure 5:**
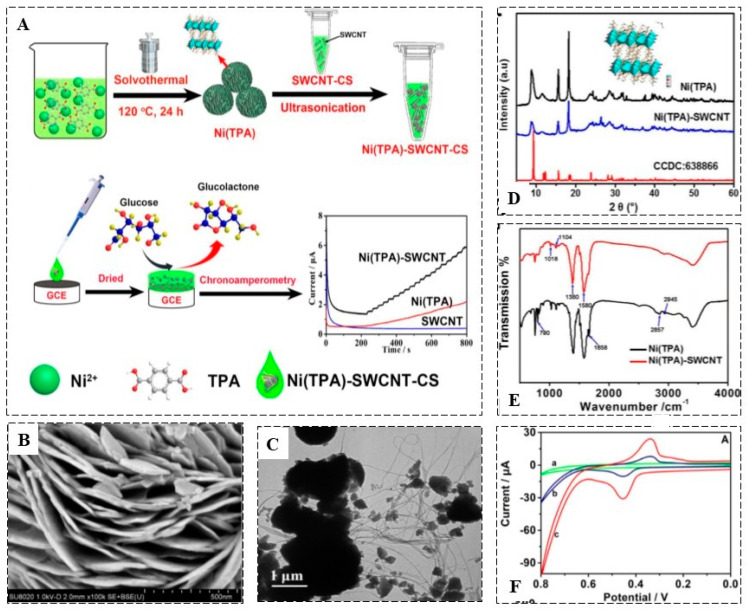
(**A**) Fabrication procedure of the Ni(TPA)-SWCNT composite and its usage as an efficient modifier agent for enzyme-free glucose detection, (**B**) SEM image of the fabricated Ni(TPA), (**C**) TEM image of the Ni(TPA)-SWCNT, (**D**) XRD patterns, (**E**) FT-IR spectra, and (**F**) Electrochemical responses of (a) GCE modified SWCNT-CS, (b) GCE modified Ni(TPA)-CS, and (c) GCE modified Ni(TPA)-SWCNT-CS. Adapted by permission from Ref. [49].

In research reported by Jia et al. [25], a flower-like nanocomposite based on carbon and nitrogen and doped with NiO (CN-NiO) was fabricated via calcination of Ni-MOF and polyaniline (PANI) at a high temperature. This composite was applied in fabricating an enzyme-free sensing platform by modifying GCE. The outcomes presented that the synthesized nanocomposite has a wide linear range according to the analytical response of glucose of a concentration 5.0 × 10^–7^–3 × 10^–3^ mol/L. The proposed assay has greater sensitivity and lower LOD of 1144 μA/mM.cm^2^ and 5.0 × 10^–7^ mol/L, respectively. Simultaneously, this electrode has high selectivity with no electrochemical signal interference with nifedipine, dopamine, ascorbic acid, and urea. Another example is the technique developed by Wang et al. [49] (Figure 5A), which is a hierarchical 3D MOF based on flower-like nickel (II)-terephthalic acid (Ni(TPA)) fabricated by a straightforward solvothermal process. Afterward, single-walled carbon nanotubes (SWCNT) and the fabricated MOF were integrated by applying the ultrasonic process to enhance the electrochemical function and the chemical stability of the composite. To study the probable application of the fabricated agent, its application as a non-enzymatic electrochemical sensor for glucose determination was investigated. Moreover, the nanocomposite has a 2.5 times higher electrochemical response for glucose determination compared with single SWCNT and fabricated Ni(TPA). The SEM image of Ni(TPA) is depicted in Figure 5B; as reported, the average thickness and the average distance among adjacent nanosheets have been evaluated to be about 25 nm and 50 nm, respectively. On the other hand, TEM images for the fabricated composite (Ni(TPA)-SWCNT) display that the microspheres are enclosed by SWCNT which have wire shapes (Figure 5C), approving the successful construction of the Ni(TPA)-SWCNT nanocomposite. As found in characterization investigations, all diffraction peaks of Ni(TPA) are preserved, proposing the retaining of the Ni(TPA) crystalline phases after doping with the SWCNT for the fabrication of nanocomposites. Furthermore, a new peak at 2θ = 26° is associated with the plane of the SWCNT, approving the existence of the carbon nanomaterial (Figure 5D). FT-IR characterization of Ni(TPA) and its composite with SWCNT are also demonstrated in Figure 5E. Electrochemical behaviors of (a) GCE modified with SWCNT-CS/, (b) GCE modified with Ni(TPA)-CS, and (c) GCE modified with Ni(TPA)-SWCNT-CS, are confirmed in Figure 5F. For glucose detection, the Ni(TPA)-SWCNT-CS modified electrode showed great analytical features, such as broad linear range, low LOD, and rapid response of 20 μM to 4.4 mM, 4.6 μM, <5 s, respectively, in addition to high selectivity. The developed sensor showed an agreement with the automatic biochemical analyzer used for glucose detection in real serum models. A correlation coefficient (r) of 0.9940 (n = 20, *p* < 0.0001) and a deviation rate of 0–6.7% were detected, which indicates the precision and potential of the sensor.

Peng et al. provided a nanocomposite of AgNPs/MOF-74 (Ni (II) as metal ions and 2,5-dioxido-1,4-benzenedicarboxylate as organic linkers) and applied it for glucose recognition through the electrochemical method. Current-time curve (I-t curve) and CV with three-electrode systems have specified the electrochemical exclusivities of the GC electrodes immobilized with the MOF-74(Ni) and Ag NPs. To sum up, the AgNPs/MOF-74(Ni) composite was set by agitation and hydrothermal treatment, and glucose oxidation was effectively promoted. The electrochemical sensing of AgNPs/MOF-74(Ni) demonstrated an LOD, sensitivity, and a broad linear range of 4.7 μΜ, 1.29 mA mM^−1^ cm^−2^, and 0.01~4 mM for glucose detection, respectively. Hence, the AgNPs/MOF-74(Ni) composite is sensible, stable, and reproducible, and has a high potential for glucose detection in biomedical applications [50]. Furthermore, Zhang et al. [51] reported an effective electrocatalyst agent by combining Ni_2_P NPs with a graphene film to prepare the Ni_2_P/G nanocomposite. The prepared composite showed a highly sensitive determination of glucose in an enzyme-free manner. By applying Ni-MOF-74 as a precursor, Ni_2_P/G presented massive exposure to active sites, even metal distribution, spatial systematic structure, and high electrocatalytic specificity toward glucose electrooxidation. In optimum conditions, a wide-range linear response from 5 μM to 1.4 mM was achieved with a 0.44 μM LOD. The sensitivities of Ni_2_P/G/GCE are 7234 μΜ, 1.29 mA mM^−1^ cm^−2^. Moreover, through applying a Ni_2_P/G modified electrode, appropriate linearity (R^2^ = 0.9897) has also been achieved in real human serum with glucose concentrations ranging from 1 mM to 8 mM. In brief, an effective electrocatalyst for non-enzymatic glucose sensors founded on MOF-derived composite has been stated. As the electrocatalytic reaction is set, Ni_2_P/G has the advantages of good electrochemical presentation towards glucose, and high specificity and selectivity for glucose determination in human serum and buffer solution. Furthermore, eco-friendly preparation and low cost have also enhanced the possibility of the application of this composite in actual models. In addition, Meng et al. [52] first applied a Co-based porous ZIF-67 as a glucose electrochemical sensor. A first-rate catalytic function against glucose oxidation was presented by the GCE modified with ZIF-67. Meanwhile, by applying a sequential deposition-reduction technique, an Ag@ZIF-67 nanocomposite was developed to modify the electrocatalytic electrode. The Ag@ZIF-67/GCE presented an enriched catalytic function against the oxidation of glucose. The electrocatalytic activity of Ag@ZIF-67 of different Ag loadings from 0% to 0.5% was presented and the sensitivity was improved by two times and a half. Here, the response period of the modified electrode decreased by more than two times. The Ag-0.5%@ZIF-67GCE displayed a good electrocatalytic presentation in a glucose concentration range of 2–1000 μM; hence, high sensitivity and low LOD of 379 μA. mM^−1^ cm^−2^ and 0.66 μM (S/N = 3), respectively, were achieved with good stability and selectivity. In research done by Li et al. [53], the hydrothermal construction of a Co-MOF nanosheet array on nickel foam from aqueous Co^2+^ and terephthalic acid as an organic ligand has been testified. High activity of glucose oxidation electrocatalysis has resulted from the Co-MOF array on Ni foam (Co-MOF/NF) in alkaline media. In the Co-MOF/NF, linear rang speedy amperometric response, low LOD, and high sensitivity of 0.001–3 mM, < 5 s, 1.3 nM (signal/noise = 3), and 10,886 μA mM^−1^ cm^−2^, respectively, have been achieved. As this study demonstrated that the material has high reproducibility and long-time stability, it may be applied for glucose recognition in fruit juice and human blood serum.

## 4. Highly Sensitive Electrochemical Sensing and Biosensing Assays for H_2_O_2_ Determination

On the other hand, the electrochemical mechanism of H_2_O_2_ determination for developing enzyme-free electrochemical determination of H_2_O_2_ is based on the electrochemical oxidation of H_2_O_2_ to H_2_O through applying the electroactive agents related to the synthesized. Table 2 demonstrates various electrochemical-based detecting platforms for the highly sensitive determination of H_2_O_2_ and some of the electrochemical research works have been described here. Huang et al. [61] developed a sensitive electrochemical sensing platform based on an active dual nanozyme amplified process by applying conductive MOF (C-MOF) nanosheets (NSs). These composites were fabricated from Au NPs and utilized in H_2_O_2_ recognition, a biomarker for cancer in living cells. Due to the high catalytic and electrical functions of Cu-HHTP-NSs and AuNPs, the provided nanocomposite immobilized electrode demonstrated well-detecting presentation to H_2_O_2_ with a low LOD and sensitivity of 5.6 nM (3σ rules) and 188.1 μA cm^−2^ mM^−1^, respectively. Moreover, the non-enzymatic biosensing platform was employed in H_2_O_2_ in a real-time manner, which has been released from dissimilar human colon cells to distinguish colon cancer cells from normal colon epithelial cells. This revealed its great role in the prompt diagnosis and management of different types of cancer. In another work by Wang et al. [62], a nanozyme sensor was developed based on an electrochemical sensing approach to recognize H_2_O_2_ concentration in living cells. By exploiting the high specific surface area of porous ZIF-67 MOF and the electrocatalytic function of Au@Pt core-shell bimetallic nanoflower structure, the non-enzyme sensor was assembled. The outcome presented that the sensor had a wide-ranging linear relationship of 0.8 μM–3 mM H_2_O_2_, while the LOD, sensitivity, and correlation coefficient were 86 nM, 24.14 μA mM^−1^ cm^−2^, and 0.9936, respectively.

**Figure 6:**
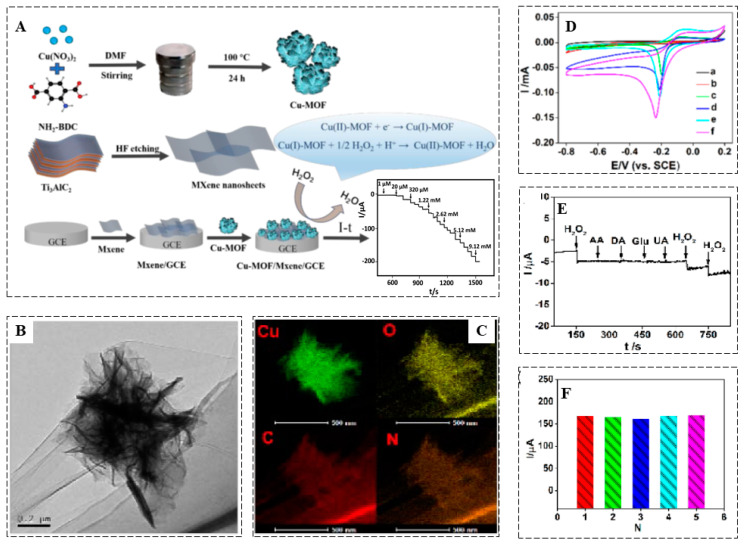
(**A**) The preparation process of 3D Cu-based flower-like MOF and MXene and the construction of the sensor based on electrochemical responses for H_2_O_2_ recognition, (**B**) Cu-MOF TEM image, (**C**) EDS elemental mapping image, (**D**) The CVs of GCE modified by MXene/(a, b), GCE modified by Cu-MOF (c, d), GCE modified with Cu-MOF and MXene (e, f), (**E**) Chronoamperometric signals of the GCE modified with Cu-MOF and MXene, CVs response of (**F**) Five independent Cu-MOF/MXene/GCE. Adapted by permission from Ref. [63].

Furthermore, Cheng et al. [63] fabricated a unique 3D flower-like Cu-MOF (benzene-1,3,5-tricarboxylate applied as a ligand) assembled with MXene to synthesize an electrochemical H_2_O_2_ sensor (Figure 6A). The catalytic function of Cu-MOF/MXene/GCE resulted in the detection due to the electrical conductivity of MXene, the nature of Cu, and the framework exclusivities of MOFs. As shown in Figure 6B, Cu-MOF is a three-dimensional flower-like structure composed of ultra-thin nanosheets, and Figure 6C depicts the EDS elemental mapping of O, N, C, and Cu of the synthesized MOF. Moreover, the electrochemical examination is performed as shown in Figure 6D, in which the CVs related to the GCE modified with MXene (a and b), GCE modified with Cu-MOF (c and d), and GCE modified with Cu-MOF and MXene (e and f) are shown. Moreover, Figure 6E demonstrates the electrochemical responses of chronoamperometric related to the GCE. Figure 6F is also related to the CV responses of (F) five independent Cu-MOF/MXene/GCE. The GCE modified with Cu-MOF and MXene presented a detection range of 1 µM to 6.12 mM using chronoamperometry with an estimated LOD of 0.35 µM at a sensing potential of −0.35 V. For now, the electrochemical sensor could be applied to recognize H_2_O_2_ in serum and milk models with pleasing outcomes. The easy operation, quick recognition, and sensitivity of the provided sensor have exhibited a noteworthy achievement for H_2_O_2_ monitoring and real-time analysis in biological models and foodstuffs.

In another work, an innovative method for Sn-based MOF (Sn-MOF) was assembled by the solvothermal process and integrated with CNT by sonochemical tools to introduce the Sn-MOF@CNT composite that was applied to H_2_O_2_ detection. The electrochemical detection of Sn-MOF@CNT demonstrated an LOD and a linear range of ~4.7 × 10^−3^ μM and 0.2 μM to 2.5 mM, respectively. This study has explored a new strategy for the deposition of CNT over Sn-MOF via a simple sonochemical methodology for successful electrochemical detection of H2O2, an approach that can be imitated for other applications [64].

**Figure 7:**
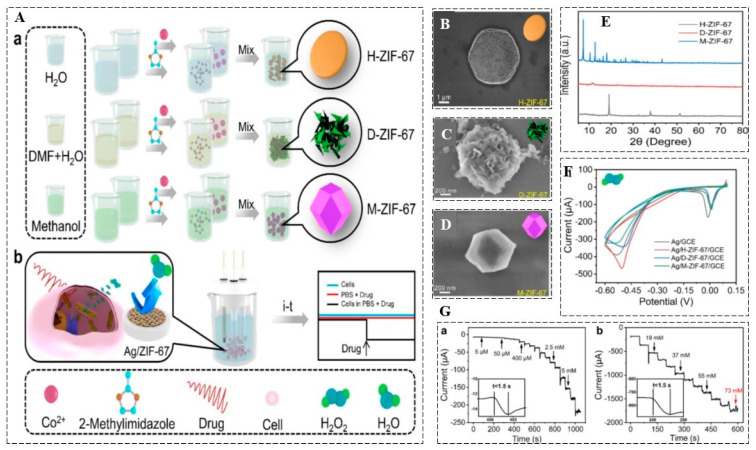
(**A**) (a) Graphic illustration of synthesis process of H-ZIF-67, D-ZIF-67, and M-ZIF-67, (b) The construction of sensing assay to detect H_2_O_2_. SEM images of (**B**) H-ZIF-67, (**C**) D-ZIF-67, and (**D**) M-ZIF-67, (**E**) XRD patterns of H-ZIF-67, D-ZIF-67, and M-ZIF-67, (**F**) CV of four diverse kinds of the electrodes, and (**G**) Amperometric signals of GCE modified with Ag/H-ZIF-67 to the following addition of (a) low and (b) high H_2_O_2_ concentration. Adapted by permission from Ref. [65].

Sun et al. [65] investigated the morphology effects and electrocatalytic function of ZIF-67 (Co zeolitic imidazolate framework-67) as a supporting substance (Figure 7A). Three kinds of ZIF-67 MOF were prepared and the morphological structures were controlled by altering the solvent by applying a polar solvent to the crystal growth and nucleation. Pure H_2_O (H-ZIF-67) and a mixed solution of DMF and H_2_O (D-ZIF-67) formed 2D ZIF-67 with microplate morphology and 2D nanosheets, respectively. However, pure methanol (M-ZIF-67) formed 3D ZIF-67 which possesses a rhombic dodecahedron structure. Then, for H_2_O_2_ reduction, Ag nanoparticles were electrodeposited on ZIF-67 modified GCE (Ag/ZIF-67/GCE). To analyze the catalytic function of GCE-modified Ag/H-ZIF-67 and GCE-modified Ag/ZIF-67, the cyclic voltammetry method was applied and demonstrated a finer electrocatalytic property than the Ag/M-ZIF-67 and Ag/D-ZIF-67 modified with GCE. SEM images of H-ZIF-67, D-ZIF-67, and M-ZIF-67 are shown in Figure 7B–D, respectively. Additionally, Figure 7E represents the XRD patterns which confirm the crystal phase of the patterns of the above-mentioned fabricated compounds. The electrochemical behaviors of the four electrodes and amperometric signals of GCE-modified Ag/H-ZIF-67 to the addition of (a) low and (b) high H_2_O_2_ concentration are illustrated in Figure 7F,G, respectively. The electrochemical H_2_O_2_ sensor presented a low detection limit of 1.1 μM, sensitivity amounts of 337.7 and 421.4 μA mM^−1^ cm^−2^, and two linear ranges of 7 mM–67 mM and 5 μM–7 mM. Furthermore, this sensor has superb stability, reproducibility, and selectivity. In addition, the sensor has been applied for real-time recognition of H_2_O_2_ from HepG2 human liver cancer cells. The research process provided a method to track the sensing potential of electrochemical sensors by changing the crystalline morphologies of supporting materials.

**Figure 8:**
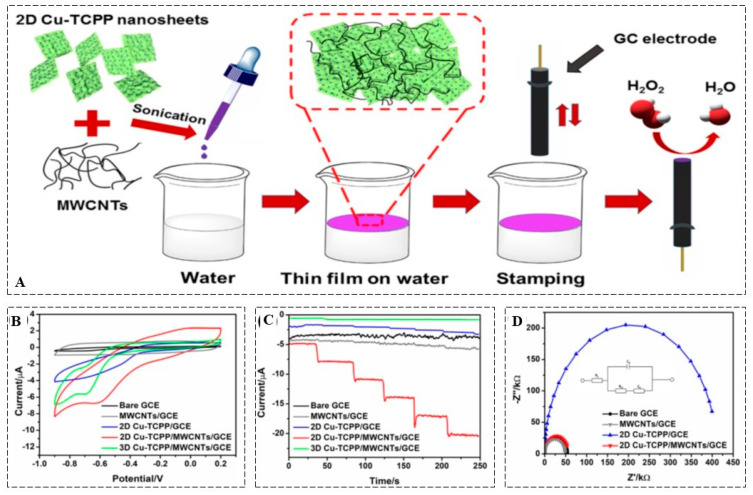
(**A**) Schematic of 2D Cu-TCPP/MWCNT preparation for H_2_O_2_ sensitive recognition, (**B**) Electrochemical signals of unmodified glassy carbon, modified MWCNTs, 2D Cu-TCPP, 2D Cu-TCPP/MWCNTs, and 3D Cu-TCPP/MWCNTs carbon electrodes, (**C**) Amperometric responses of bare glassy carbon, and GC electrodes modified with MWCNTs, 2D Cu-TCPP, 2D Cu-TCPP/MWCNTs, and 3D Cu-TCPP/MWCNTs, and (**D**) EIS spectra of bare glassy carbon, GC modified MWCNTs, 2D Cu-TCPP, and 2D Cu-TCPP/MWCNTs electrodes. Adapted by permission from Ref. [66].

Wang et al. [66] synthesized 2D MOF nanosheets that combined with MWCNT films and immobilized them on electrodes as an electrochemical component for H_2_O_2_ detection. Two-dimensional Cu-TCPP/MWCNTs/GCE showed 0.70 μM LOD (S/N = 3), a sensitivity of 157 μA cm^−2^ mM^−1^, a short response time of fewer than 3 s, and a linear range from 0.001 to 8.159 mM. The occurrence of MWCNTs with high electrical conductivity has enhanced the sensitivity of composite films in comparison with the original 2D MOF nanosheets. Figure 8A represents the graphic steps of the 2D Cu-TCPP (copper-tetrakis (4-carboxyphenyl) porphyrin)/MWCNTs preparation process. Electrochemical behavior of the bare GCE, GC modified with MWCNTs, GC modified with two-dimensional Cu-TCPP, GC modified with two-dimensional Cu-TCPP/MWCNTs, and GC modified with three-dimensional Cu-TCPP/MWCNTs electrodes are shown in Figure 8B. Moreover, Figure 8C shows the amperometric signals of the bare glassy carbon, GC-modified electrode with MWCNTs, GC-modified electrode with two-dimensional Cu-TCPP, two-dimensional Cu-TCPP/MWCNTs/GC, and GC-modified electrode with three-dimensional Cu-TCPP/MWCNTs. Moreover, impedance spectra of the bare glassy carbon, GC-modified MWCNTs, 2D Cu-TCPP, and 2D Cu-TCPP/MWCNTs electrodes have been indicated in Figure 8D. The sensitive, selective straightforward, and stable H_2_O_2_ sensors are suitable for H_2_O_2_ detection in actual models; beer and serum are examples.

In another work by Menon et al. [12], a copper-MOF fabricated via solvothermal conditions was presented to show a basic peroxidase-like function that has been electrochemically and spectro-photometrically developed. To sense glucose, the peroxidase mimics could be combined with the enzymatic oxidation of glucose. The recognition of glucose and H_2_O_2_ through applying Cu-MOF was highly selective to the common interfering species. The amperometric response presented the efficacy of Cu-MOF modified electrode towards H_2_O_2_ with sensitivity, linear range, and an LOD of 263 μA mM^−1^ cm^−2^, 25 μM −30,000 μM, and 25 μM (S/N = 3), respectively, at an applied voltage of 0.50 V.

## 5. Conclusions and Future Outlooks

This review paper has presented a summary of the electrochemical-based investigations of glucose and H_2_O_2_ by applying MOF-based compounds. The MOF-based nanomaterials are of great importance and are experiencing much demand for developing glucose and H_2_O_2_ sensors due to their remarkable fabrication technologies, widespread and comprehensive study, and high sensitivity, as well as application of innovative electrode materials. However, attaining higher selectivity, fast response time, sensitivity, miniaturization, stability, reliable accuracy and precision, and automatic systems have always been significant challenges in designing a new sensing platform. In this regard, only a few sensing platforms apply to in vivo determinations and there are still requirements for developing methods for the detection of glucose and H_2_O_2_ in biological applications. We have comprehensively reviewed the recent trends in the design of glucose and H_2_O_2_ sensors by using MOF-based nanomaterials. These compounds are porous organic–inorganic hybrid materials with strong adsorption capacity, excellent catalytic ability, large specific surface area, and efficient loading capability. Based on this, to detect glucose and H_2_O_2_, MOF-based electrochemical approaches have been provided as sensing tools with exceptional selectivity and responsiveness using progressive nanostructures and smaller devices. Additional progress in these methods is probable through integrating various approaches to overcome the limitations. The upcoming research work aims to emphasize progressing low-complexity techniques for amateurs. As demonstrated, a wide range of enzyme- and non-enzyme-based biosensors can be effectively established for the sensitive determination of both glucose and H_2_O_2_. In addition, enzyme-free-based biosensors have been verified to be the most promising ones. As a prospect, the application of immunosensors, which utilize antigens or antibodies as specific biosensors for the recognition of glucose and H_2_O_2_, is promising. This technique is more specific than the enzyme-based biosensing one. Moreover, applying novel MOFs with innovative synthesized techniques unlocks new potentials in sensing and biosensing approaches.

## Figures and Tables

**Table 1:** Various electrochemical-based detecting platforms for sensitive determination of glucose.

Electrode Material	Linear Range/µM	Detection Limit/μM	Sensitivity/µA mM^−1^cm^−2^	Reference
NiMn-LDH-MOF/GCE	4.9–2200	0.87	849	[41]
Ni-MOF/GCE	1–1600	0.76	2859.95	[42]
2D CuCo-MOF/CFP	10–1200	0.12	68.61	[1]
Bimetallic NCNT MOF CoCu nanostructure/GCE	2600–5500	0.15	10.27	[43]
Ni/Co(HHTP)MOF/CC	0.3–2312	0.1	3250	[16]
CC@MOF-74(NiO)@NiCo LDH	10–1100	0.288	1699	[44]
conductive Ni-MOF	1–8000	0.66	21,744	[45]
core-shell UiO-67@Ni-MOF/GCE	5–3900	0.98	-	[46]
CoCu-MOF/Cu_2_O/CFE	1–1070	0.72	11.916	[47]
FeBDC-derived Fe_3_O_4_ MOF/GCE	0–9000	15.70	4.67	[48]
Ni-MOF/PANI-Derived CN-Doped NiO/GCE	50–3000	50	1144	[25]
Ni(TPA)-SWCNT-CS/GCE	20–4400	4.6	-	[49]
AgNPs/MOF-74(Ni)/GCE	10–4000	4.7	1290	[50]
Ni2P/G/GCE	5–1400	0.44	7234	[51]
Ag@ZIF-67/GCE	2–1000	0.66	379	[52]
Co-MOF/NF	1–3000	0.0013	10,886	[53]
GCE/ZIF-8-Au NP-GOx	0.05–0.45	5	-	[54]
Ni-MOF/Ni/NiO/C	4–5664	0.8	367.45	[55]
IL/GOx/Ag@Zn-TSA-CPE	2.0–1022	0.8	-	[56]
GOx/MOFs/PtNPS	5–1400	5	-	[57]
Iridium(III)-MOFs	50–5000	10	-	[58]
GCE/Zn-MOF-74-rGO Pt NPs-GOx	6–6000	1.8	64.51	[59]
Cu-MOF [Cu_3_(btc)_2_]	0.125–2250	-	549	[60]

**Table 2:** Various electrochemical-based detecting platforms for sensitive determination of H_2_O_2_.

Electrode Material	Linear Range/µM	Detection Limit/μM	Sensitivity/µA mM^−1^cm^−2^	Reference
Cu-HHTP-NSs and Au-NPs	-	0.0056	188.1	[61]
MOF-Pt@Au/GCE	0.8–3000	0.086	24.14	[62]
Cu-MOF/MXene/GCE	1–6120	0.35	-	[63]
Sn-MOF@CNT/Au	0.2–2500	0.0047	-	[64]
Ag/H-ZIF-67/GCE	5–7000, 7000−67,000	1.1	421.4 and 337.7	[65]
2D Cu-TCPP/MWCNTs/GCE	1–8159	0.70	157	[66]
Cu-MOF/GCE	25–30,000	25	263	[12]
UiO-66-NH_2_@P(ANI-co-ANA)/GCE	25–500	0.6	1396.1	[17]
AuNPs-NH_2_/Cu-MOF/GCE	5–850	1.2	-	[67]
MNPs@Y^−1^, 4-NDC-MOF/ERGO/GCE	4–11,000	0.18	8.80	[28]
3D-KSC/PCN-333 (Al)@MP-11	0.387–1725	0.127	168	[68]
Cu-hemin MOFs/CS-rGO	0.065–410	0.019	14.5	[69]
CuxO NPs@ZIF-8	1.5–21,442	0.15	178	[70]
ZIF-67/Ti@TiO_2_/CdS	5–5000, 5000–14,000	1.11	-	[71]
Co(pbda)(4,4-bpy).2H_2_O]n	5–9000	3.76	83.1	[26]
Cu-MOF-GN-3/GCE	10–11,180	2	57.73	[72]
CuMOF/GCE	0.9–990	1	78.22	[73]
ZIF-67 NWs/CF	10–1050	1.4	-	[74]
CuMOF/CPE	1–900	1	76	[75]
ZIF-67/MWCNTs/CPE	20–430	2.46	-	[76]

## Data Availability

The data presented in this study are available on request from the corresponding author.
